# High-flow nasal cannula versus continuous positive airway pressure for noninvasive respiratory support in children with bronchiolitis: a meta-analysis of randomized controlled trials

**DOI:** 10.3389/fped.2026.1892131

**Published:** 2026-08-05

**Authors:** Boying Wu, Zhe Zhang, Yajiao Pang

**Affiliations:** Department of Pediatrics, The Affiliated People’s Hospital of Ningbo University, Ningbo, China

**Keywords:** bronchiolitis, high-flow nasal cannula, meta-analysis, positive airway pressure, treatment failure

## Abstract

**Background:**

High-flow nasal cannula (HFNC) and continuous positive airway pressure (CPAP) are increasingly used for noninvasive respiratory support in children with bronchiolitis, but their comparative efficacy and safety remain uncertain. This meta-analysis aimed to compare HFNC and CPAP in children with bronchiolitis based on evidence from randomized controlled trials (RCTs).

**Methods:**

PubMed, Cochrane Library, Embase, Web of Science, Wanfang, and CNKI were systematically searched for RCTs comparing HFNC and CPAP in children with bronchiolitis. The primary outcome was treatment failure. Secondary outcomes included invasive mechanical ventilation (MV) and adverse events (AEs). Risk ratios (RRs) with 95% confidence intervals (CIs) were pooled using a random-effects model.

**Results:**

Ten RCTs involving 965 children were included. Overall, HFNC was not associated with a significantly different risk of treatment failure compared with CPAP (RR: 0.80, 95% CI: 0.50–1.29, *p* = 0.37; moderate-certainty evidence). Subgroup analyses suggested that HFNC was associated with a lower risk of treatment failure in children treated in pediatric wards, in studies with mean age >3 months, and in those with mean body weight >6 kg. The rate of invasive MV was also not significantly different between groups (RR: 0.88, 95% CI: 0.61–1.29, *p* = 0.51; low-certainty evidence). Howevnker, HFNC was associated with a significantly lower incidence of AEs compared with CPAP (RR: 0.35, 95% CI: 0.22–0.57, *p* < 0.001; low-certainty evidence).

**Conclusions:**

HFNC and CPAP appear to provide comparable efficacy for noninvasive respiratory support in children with bronchiolitis, while HFNC may reduce device-related AEs. Subgroup and meta-regression analyses further suggested that the relative efficacy of HFNC may be influenced by patient age and body weight, with more favorable effects observed in older and heavier children.

**Systematic Review Registration:**

https://www.crd.york.ac.uk/PROSPERO/view/CRD420261404713, PROSPERO CRD420261404713.

## Introduction

Bronchiolitis is one of the most common lower respiratory tract infections and a leading cause of hospitalization in infants and young children worldwide, particularly during the first two years of life (1, 2). Respiratory syncytial virus (RSV) is the predominant causative pathogen, and severe cases are characterized by airway inflammation, mucus plugging, hypoxemia, and increased work of breathing, which may progress to respiratory failure requiring pediatric intensive care unit (PICU) admission (3, 4). Although the management of bronchiolitis is largely supportive, noninvasive respiratory support has become increasingly important for children with moderate-to-severe disease because it may improve oxygenation, reduce respiratory effort, and avoid invasive mechanical ventilation (MV) and its related complications (5, 6). Accordingly, modalities such as high-flow nasal cannula (HFNC) and continuous positive airway pressure (CPAP) have been increasingly adopted in pediatric wards and PICUs for the respiratory management of bronchiolitis (5, 6).

HFNC delivers heated and humidified gas at high flow rates through nasal cannulas, which can reduce nasopharyngeal dead space, improve mucociliary clearance, and generate a certain degree of positive end-expiratory pressure (PEEP) (7, 8). HFNC is generally considered easier to administer, better tolerated, and more comfortable for children than CPAP because it does not require a tightly fitted interface (9). In contrast, CPAP provides continuous positive airway pressure throughout the respiratory cycle, thereby improving functional residual capacity, preventing airway collapse, and reducing inspiratory work of breathing (10, 11). Although CPAP may offer more stable airway pressure support in severe bronchiolitis, it is also associated with greater patient discomfort, nasal trauma, and the need for closer monitoring (12). Therefore, determining the optimal noninvasive respiratory support strategy for bronchiolitis remains clinically important.

Several previous systematic reviews and meta-analyses have compared HFNC and CPAP in children with bronchiolitis, but the results remain inconsistent (13–16). Early meta-analyses in 2022 including only five randomized controlled trials (RCTs) reported that CPAP was associated with a lower risk of treatment failure than HFNC, while no significant difference was observed in invasive MV (15, 16). Similarly, a recent 2026 meta-analysis incorporating both prospective and retrospective studies also suggested lower treatment failure rates with CPAP but included substantial clinical heterogeneity and nonrandomized evidence (13). In contrast, another recent meta-analysis including six RCTs found no statistically significant difference in treatment failure between HFNC and CPAP, although heterogeneity was considerable and sensitivity analysis yielded conflicting results (14). These previous studies were limited by relatively small sample sizes, inclusion of retrospective studies, variable definitions of treatment failure, and limited exploration of potential effect modifiers such as age, body weight, and clinical setting. Therefore, we performed an updated systematic review and meta-analysis of RCTs to compare the effects of HFNC and CPAP on treatment failure, invasive MV, and adverse events (AEs) in children with bronchiolitis, while also exploring potential sources of heterogeneity through subgroup and meta-regression analyses.

## Methods

This systematic review and meta-analysis followed established methodological guidance from the PRISMA (Preferred Reporting Items for Systematic Reviews and Meta-Analyses) reporting framework (17) and the Cochrane Handbook for Systematic Reviews of Interventions (18). The review protocol was registered prospectively with the PROSPERO international prospective register of systematic reviews (CRD420261404713). No substantive deviations from the registered PROSPERO protocol occurred during the conduct of this review.

### Study inclusion and exclusion criteria

Eligible studies were selected based on predefined criteria structured according to the PICOS framework.

P (Population): Studies enrolling children with bronchiolitis, including infants and pediatric patients diagnosed with acute viral bronchiolitis based on clinical criteria, physician diagnosis, or institutional guidelines. Studies conducted in emergency departments, pediatric wards, high-dependency units, or pediatric intensive care units (PICU) were eligible. Studies including mixed respiratory diseases were included only if data for bronchiolitis patients could be extracted separately.

I (Intervention): HFNC therapy, including heated humidified high-flow nasal cannula oxygen therapy delivered through dedicated HFNC systems.

C (Comparator): CPAP-based respiratory support, including ventilator CPAP, nasal CPAP (nCPAP), and bubble CPAP (bCPAP).

O (Outcomes): Studies reporting at least one of the following outcomes were included: (1) treatment failure, defined according to the criteria used in the individual studies, including worsening respiratory distress, persistent hypoxemia, escalation of respiratory support, transfer to intensive care, or clinician-defined failure requiring additional intervention; (2) intubation/invasive MV rate, defined as the requirement for endotracheal intubation and invasive MV during hospitalization; (3) all-cause mortality during hospitalization; and (4) overall AEs, such as nasal trauma, abdominal distension, air leak syndrome, pneumothorax, skin injury, discomfort, or intolerance.

S (Study design): Only randomized controlled trials (RCTs) with parallel groups were included.

Studies were excluded if they: (1) included adults or mixed pediatric populations without separately extractable bronchiolitis data; (2) compared HFNC with conventional oxygen therapy rather than CPAP; (3) were observational studies, retrospective studies, cohort studies, case-control studies, case series, case reports, reviews, editorials, letters, study protocols, or animal studies; (4) were physiological crossover studies or short-term experimental studies without clinically relevant outcomes; (5) lacked sufficient data for outcome extraction; or (6) included duplicate or overlapping populations, in which case the one with the largest sample size was included.

### Database search

PubMed, Cochrane Library, Embase, Web of Science, Wanfang, and China National Knowledge Infrastructure (CNKI) were systematically searched using the combined terms including: (1) “bronchiolitis” OR “viral bronchiolitis” OR “RSV” OR “respiratory syncytial virus”; (2) “high-flow nasal cannula” OR “high flow nasal cannula” OR “HFNC” OR “high-flow oxygen” OR “HHHFNC” OR “HHHF”; (3) “CPAP” OR “nasal CPAP” OR “bubble CPAP” OR “NIV” OR “NIPPV” OR “noninvasive ventilation” OR “non-invasive ventilation” OR “noninvasive positive pressure ventilation”; and (4) “randomized” OR “randomised” OR “randomly” OR “trial” OR “RCT” OR “control” OR “controlled” OR “allocated” OR “assigned”. We considered only peer-reviewed, English or Chinese language full-text studies conducted in human participants. In addition, the bibliographies of relevant reviews and eligible articles were manually screened to identify any additional records. The final search update was performed on March 23, 2026, and the detailed database-specific search strategies are provided in Supplementary File 1.

### Study quality evaluation

Two reviewers independently performed the literature search, screened the retrieved records for eligibility, extracted relevant data, and evaluated study quality. Any disagreements were resolved through discussion with the third author until consensus was reached among the authors. The methodological quality of the included randomized controlled trials was evaluated using the Cochrane Risk of Bias tool (RoB 2.0), which examines potential bias across five domains: the randomization process, deviations from intended interventions, missing outcome data, measurement of outcomes, and selection of the reported results (18). Each domain was rated as low risk, some concerns, or high risk of bias, and these judgments were subsequently integrated to generate an overall risk-of-bias assessment for each study.

### Data extraction

Extracted data included general study characteristics (first author, publication year, and country), study design (double-blind, single-blind, or open-label), clinical setting [pediatric ward or pediatric intensive care unit (PICU)], participant characteristics (number of children included, mean age, sex distribution, mean body weight, proportion of children born prematurely, and proportion of children with RSV positivity), details of HFNC therapy (HFNC device/system, initial flow rate, maximum flow rate, FiO_2_ protocol, use of heated humidified systems, and weaning protocol), details of CPAP therapy (CPAP type, initial pressure, interface type including nasal prongs, mask, or helmet, and weaning protocol), outcomes reported, definitions of treatment failure, and the number of children with treatment failure in each study.

### Statistical analysis

The effects of HFNC compared with CPAP on the primary outcome of treatment failure and the secondary outcomes of invasive MV, all-cause mortality, and overall AEs in children with bronchiolitis were summarized as risk ratios (RRs) with corresponding 95% confidence intervals (CIs). Between-study heterogeneity was assessed using Cochran's *Q* test and quantified with the *I*^2^ statistic. *I*^2^ values of <25%, 25%–75%, and > 75% were considered to indicate low, moderate, and high heterogeneity, respectively (19). Pooled estimates were generated using a random-effects model to account for anticipated clinical and methodological diversity among trials (18). The stability of the findings was evaluated through leave-one-out analyses (18). In addition, an additional sensitivity analysis was performed by restricting the primary outcome analysis to studies in which treatment failure was defined primarily using prespecified physiological thresholds and predefined escalation criteria. For the primary outcome, prespecified subgroup analyses were performed according to clinical setting (PICU vs. pediatric ward), mean age of the children, mean body weight, and study quality based on the RoB 2.0 assessment (low-risk studies vs. studies with some concerns). Medians of continuous variables were used as cutoff values for subgroup analyses to ensure a balanced distribution of studies across subgroups. In addition, univariate meta-regression analyses were also performed to explore the potential influence of study characteristics on the results of the meta-analysis, such as the sample size, mean age of the children, proportion of boys, and mean body weight. Potential publication bias was assessed using funnel plot inspection and Egger's regression test (20). Statistical significance was defined as a two-sided *p* value < 0.05. All analyses were carried out using RevMan (version 5.3, Cochrane Collaboration, Oxford, UK) and Stata (version 17.0, StataCorp, College Station, TX, USA) software.

### Certainty of evidence

Two reviewers independently assessed the certainty of evidence using the GRADE (Grading of Recommendations, Assessment, Development and Evaluation) approach, which evaluates the level of confidence in the results based on several domains, including risk of bias, inconsistency, indirectness, imprecision, and potential publication bias (21). The overall certainty for each outcome was classified as high, moderate, low, or very low. Any disagreements between reviewers were resolved through discussion until a consensus was reached.

## Results

### Literature search

**Figure 1:**
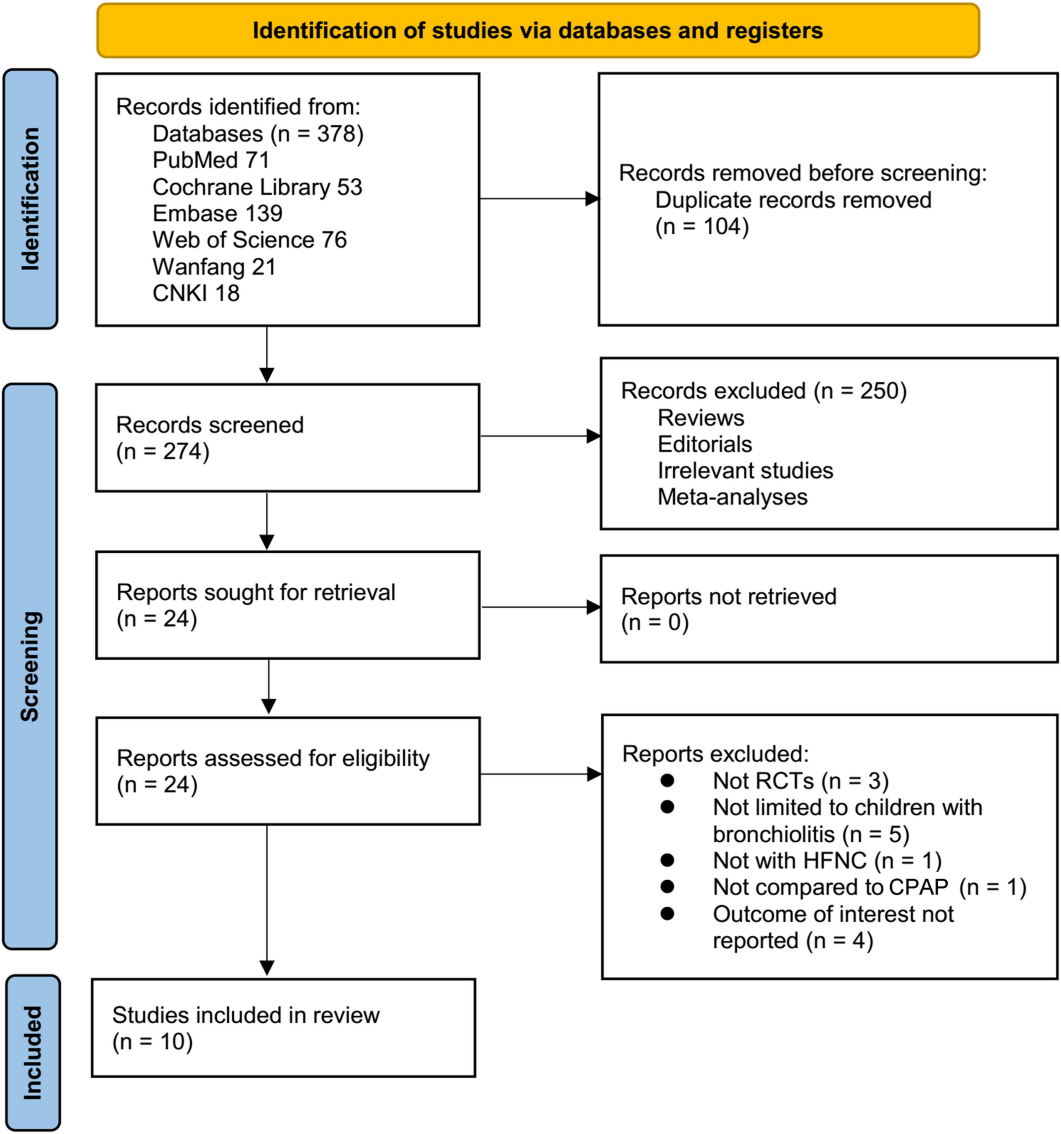
Flowchart for the literature search and study inclusion.

Figure 1 illustrates the study selection process for this meta-analysis. The database search initially identified 378 records, of which 274 remained after removing duplicates. Screening of titles and abstracts led to the exclusion of 250 articles that were not relevant to the study objective. The full texts of the remaining 24 articles were then reviewed in detail, and 14 were excluded for the reasons summarized in Figure 1. Ultimately, 10 RCTs (22–31) met the eligibility criteria and were included in the quantitative synthesis.

### Characteristics of the included studies

The main characteristics of the included RCTs are summarized in Table 1. This meta-analysis included 10 RCTs (22–31) published between 2017 and 2024, conducted across multiple countries, including France, China, India, Denmark, Brazil, and Tunisia. A total of 965 children with bronchiolitis were enrolled. The included studies were conducted in pediatric wards (26–28, 30) or PICUs (22–25, 29, 31), and all enrolled children with moderate-to-severe bronchiolitis requiring noninvasive respiratory support. Sample sizes ranged from 31 to 255 children. The mean age of participants varied from 1.3 to 14.1 months, and the proportion of boys ranged from 45.2% to 66.1% among studies reporting these data. Mean body weight ranged from 4.1 to 9.4 kg. The proportion of children born prematurely ranged from 0% to 18.3%, while RSV positivity ranged from 21.2% to 90.0% in studies reporting virological data. All included studies compared HFNC with CPAP-based respiratory support. HFNC interventions generally involved heated humidified systems, with most studies using initial flow rates of approximately 2 L/kg/min or protocolized flow escalation strategies. FiO_2_ was commonly titrated to maintain target SpO_2_ levels between 92% and 97%. CPAP comparators mainly consisted of nCPAP, while one study used bubble CPAP (31). Initial CPAP pressures generally ranged from 4 to 7 cmH_2_O. Nasal prongs or nasal masks were the most commonly used interfaces. Treatment failure was reported in all included studies, whereas invasive MV and AEs were reported in seven (22–26, 29, 31) and six (22–24, 29–31) studies, respectively. Definitions of treatment failure varied across studies but generally included worsening respiratory distress, persistent hypoxemia despite respiratory support escalation, increasing respiratory support requirements, transfer to intensive care, or the need for invasive MV. Only one large multicenter study reported all-cause mortality (29), in which no deaths occurred among children treated with HFNC, whereas one death was reported among 125 children treated with CPAP. However, the difference was not statistically significant (0/130 vs. 1/125, *p* = 0.49).

**Table 1:** Characteristics of the included studies.

Study	Country	Design	Study setting	No. of children included	Mean age (months)	Boy (%)	Mean BW (kg)	Prematurity (%)	RSV positivity (%)	Details of HFNC	Details of CPAP	Outcomes reported	Definition of treatment failure	No. of children with treatment failure
Milesi 2017	France	R, OL	PICU	142	1.3	NR	4.1	18.3	88.0	Device: Optiflow (Fisher & Paykel); Initial flow: 2 L/kg/min; Maximum flow: Not specified (fixed at 2 L/kg/min); FiO_2_ protocol: Titrated to SpO_2_ 94%–97%; Heated humidified: Yes, 37 °C auto-set; Weaning protocol: Not specified	Type: nCPAP (Infant Flow Ventilator or FlexiTrunk infant interface connected to ventilator CPAP); Initial pressure: 7 cmH_2_O; Interface: Nasal; Weaning protocol: Not specified	Treatment failure; Invasive MV rate; and adverse events	≥1 of: (1) 1-point increase in mWCAS from baseline; (2) RR rise >10 bpm from baseline with RR >60 bpm; (3) 1-point increase in EDIN score from baseline with EDIN >4 despite hydroxyzine; (4) >2 severe apnea episodes/hour requiring bag-mask ventilation despite caffeine loading	58
Ji 2017	China	R, OL	PICU	32	3.4	50.0	6.0	NR	NR	Device: HFNC (heated humidified high-flow nasal cannula); Initial flow rate: 2 L/min; Maximum flow rate: 25 L/min (failure criterion); FiO_2_ protocol: Titrated to maintain SpO_2_ > 92% (FiO_2_ increased if SpO_2_ < 92% despite 300 mL/L oxygen); Heated humidified system: Yes, 34 °C, pressure 32 cmH_2_O/L; Weaning protocol: Discontinue when FiO_2_ ≤ 300 mL/L or flow ≤4 L/min maintains SpO_2_ 92%–98%, then observe SpO_2_ > 92% for >4 h	Type: nCPAP (nasal continuous positive airway pressure); Initial pressure: 4–6 cmH_2_O; Maximum pressure: 10 cmH_2_O (failure criterion); Interface: Nasal prongs; Weaning protocol: Discontinue when pressure 4 cmH_2_O maintains SpO_2_ 92%–98%, then observe SpO_2_ > 92% for >4 h	Treatment failure; Invasive MV rate; and adverse events	≥1 of: SpO_2_ < 88% despite FiO_2_ > 600 mL/L OR flow ≥25 L/min (HFNC) or pressure ≥10 cmH_2_O (nCPAP) with SpO_2_ < 92%; severe metabolic acidosis (BE < −10 mmol/L); severe respiratory acidosis (PaCO_2_ > 65 mmHg)	4
Sarkar 2018	India	R, OL	PICU	31	3.4	45.2	NR	NR	NR	Device: AIRVO™2 (Fisher & Paykel); Initial flow rate: 2 L/kg/min (for ≤10 kg), for >10 kg: 2 L/kg/min for first 10 kg + 0.5 L/kg/min per kg above; Maximum flow rate: Not specified; FiO_2_ protocol: 0.4 at initiation, then titrated to maintain SpO_2_ ≥ 94%; Heated humidified system: Yes (hot humidified, 37 °C, 100% relative humidity); Weaning protocol: Not specified	Type: nCPAP via nasal mask (SERVO-i®, Maquet); Initial pressure: 4 cmH_2_O; Maximum pressure: 8 cmH_2_O; Interface: Nasal mask or nasal prong; Weaning protocol: Not specified	Treatment failure; Invasive MV rate; and adverse events	HR/RR unchanged/increased; required FiO_2_ > 60% for nCPAP with PEEP >8 cmH_2_O OR FiO_2_ > 60% for HFNC with max flow rate to maintain SpO_2_ > 94%; no improvement/increase in RDAI score	2
Vahlkvist 2020	Denmark	R, OL	Pediatric ward	50	2.5	60.0	5.2	NR	90.0	Device: Not specified (likely Fisher & Paykel); Initial flow rate: Not specified; Maximum flow rate: Not specified; FiO_2_ protocol: Titrated to maintain SpO_2_ ≥ 94%; Heated humidified system: Yes (standard HFNC); Weaning protocol: Breaks when condition improved as attempt to discontinue	Type: CPAP (not specified as nCPAP/bubble/ventilator); Initial pressure: Not specified; Interface: Nasal; Weaning protocol: Breaks when condition improved as attempt to discontinue	Treatment failure	Assessed by physician in charge—decision to switch system due to disease progression or poor system tolerance, OR transfer to PICU with/without mechanical ventilation	6
Li 2020	China	R, OL	Pediatric ward	90	5.2	48.9	8.8	NR	NR	Device: Heated humidified HFNC; Initial flow rate: 4–6 × minute ventilation (specific L/min not given); Maximum flow rate: Not specified; FiO_2_ protocol: 30%–50%, titrated to maintain TeSO_2_ and clinical signs; Heated humidified system: Yes; Weaning protocol: Decrease parameters when condition improves	Type: nCPAP; Initial pressure: 3–5 cmH_2_O; Maximum pressure: Not specified; Interface: Nasal; Flow rate: 3 × minute ventilation; FiO_2_: 30%–50%; Weaning protocol: Decrease parameters when condition improves	Treatment failure; and Invasive MV rate	Unsatisfactory response to HFNC or nCPAP oxygen therapy requiring endotracheal intubation and mechanical ventilation	4
Cesar 2020	Brazil	R, OL	PICU	63	3.0	NR	5.7	NR	88.9	Device: Precision Flow (Vapotherm); Initial flow rate: Not specified (titrated up to max); Maximum flow rate: 1.5 L/kg/min; FiO_2_ protocol: Adjusted to maintain SpO_2_ > 93%; Heated humidified system: Yes (hollow fiber heated humidified system); Weaning protocol: Continued until successfully weaned to simple oxygen cannula	Type: CPAP via nasal prongs (Hudson RCI/Teleflex) from Drager Evita 4 ventilator; Initial pressure: 6 cmH_2_O (fixed for all patients); Maximum pressure: Not applicable (fixed at 6 cmH_2_O); Interface: Soft anatomically curved nasal prongs; Weaning protocol: Continued until successfully weaned to simple oxygen cannula	Treatment failure; and Invasive MV rate	Need to escalate support to noninvasive bilevel pressure ventilation (BiPAP) or endotracheal intubation (no crossover between groups)	23
Zhang 2020	China	R, OL	Pediatric ward	100	14.1	56.0	NR	NR	NR	Device: HFNC (heated humidified high-flow nasal cannula); Initial flow rate: 4–6 × minute ventilation (specific L/min not given); Maximum flow rate: Not specified; FiO_2_ protocol: 30%–60%; Temperature: 32–37 °C; Heated humidified system: Yes; Weaning protocol: Not specified	Type: nCPAP (nasal continuous positive airway pressure); Initial pressure: 4–6 cmH_2_O; Interface: Nasal; FiO_2_: 50%; Weaning protocol: Not specified	Treatment failure	Unchanged or worsening clinical symptom scores necessitating escalation of respiratory support	15
Qi 2021	China	R, OL	Pediatric ward	84	10.0	55.9	9.4	0.0	NR	Device: German Stephen pediatric noninvasive ventilator with high-flow humidified nasal cannula; Initial flow rate: 5 L/min; Maximum flow rate: 6 L/min (if TeSO_2_ < 95% after 60 min); FiO_2_ protocol: 30% initial; Temperature: Heated and humidified (standard); Weaning protocol: Not specified	Type: nCPAP (German Stephen nasal continuous positive airway pressure system); Initial pressure: Not specified (adjusted based on blood gas to maintain PaCO_2_ 40–50 mmHg, PaO_2_ 60–80 mmHg, TeSO_2_ > 95%); Interface: Nasal; Weaning protocol: Not specified	Treatment failure; and adverse events	Failure to achieve treatment effect criteria within 2 days, necessitating escalation of respiratory support	10
Borgi 2021	Tunisia	R, OL	PICU	255	1.7	59.2	4.5	9.0	45.1	Device: Fisher & Paykel Healthcare HFNC system; Cannula sizes: Neonatal BC2435 (max 6 L/min), Infant BC2745 (max 7 L/min), Intermediate BC2755 (max 7 L/min); Initial flow rate: Maximum for cannula size; FiO_2_ protocol: Titrated to maintain SpO_2_ 94%; Temperature: 37 °C constant; Heated humidified system: Yes; Weaning protocol: Decrease flow by 1 L/min every 2 h when FiO_2_ < 30% to reach 2 L/min, then wean if FiO_2_ < 30% and flow ≤2 L/min for ≥6 h; Failure criteria: FiO_2_ > 60% to maintain SpO_2_ ≤ 90% or increasing work of breathing	Type: CPAP via neonatal ventilator (Babylog 8000); Initial pressure: 6 cmH_2_O; Maximum pressure: 8 cmH_2_O; Interface: Nasal mask or nasal prongs (physician discretion); Weaning protocol: Decrease PEEP by 1 cmH_2_O every 6 h from optimal PEEP when FiO_2_ < 30% and no increased WOB; wean from CPAP if PEEP <6 cmH_2_O and FiO_2_ < 30% for ≥6 h	Treatment failure; Invasive MV rate; all-cause mortality; and adverse events	Need for escalation of care: FiO_2_ > 60% to maintain SpO_2_ ≤ 90% OR increasing work of breathing	101
Maya 2024	India	R, OL	PICU	118	5.5	66.1	6.7	10.0	21.2	Device: AIRVO2 (Fisher & Paykel Healthcare, New Zealand); Initial flow rate: 2 L/kg/min (if ≤10 kg); 20 L/min + 0.5 L/kg/min for each kg >10 (none met this); Maximum flow rate: As above; FiO_2_ protocol: Starting FiO_2_ 0.60, titrated to SpO_2_ 94%–97%; Temperature: Heated/humidified (standard); Weaning protocol: Wean FiO_2_ first to ≤0.25, then flow to 4 L/min, maintain 4 h, then nasal prong 1 L/min for 2 h, then stop	Type: Nasal prong bubble CPAP (b-CPAP)—underwater seal system; Initial pressure: 5 cmH_2_O; Maximum pressure: 7 cmH_2_O; Interface: Nasal prong; Weaning protocol: Wean FiO_2_ first, then pressure to 5 cmH_2_O, maintain 4 h, then nasal prong 1 L/min for 2 h, then stop	Treatment failure; Invasive MV rate; and adverse events	Any of: 1) 1-point increase in mWCAS from baseline; 2) RR rise >10 breaths/min from baseline; 3) escalation in respiratory support technique	39

NR, not reported; R, randomized; OL, open-label; PICU, pediatric intensive care unit; BW, body weight; RSV, respiratory syncytial virus; HFNC, high-flow nasal cannula; CPAP, continuous positive airway pressure; nCPAP, nasal continuous positive airway pressure; b-CPAP, bubble continuous positive airway pressure; MV, mechanical ventilation; FiO_2_, fraction of inspired oxygen; SpO_2_, peripheral oxygen saturation; TeSO_2_, transcutaneous oxygen saturation; RR, respiratory rate; HR, heart rate; PEEP, positive end-expiratory pressure; PaCO_2_, partial pressure of arterial carbon dioxide; PaO_2_, partial pressure of arterial oxygen; BE, base excess; bpm, breaths per minute; mWCAS, modified Wood's Clinical Asthma Score; EDIN, Échelle Douleur Inconfort Nouveau-Né scale; RDAI, Respiratory Distress Assessment Instrument; NIV, noninvasive ventilation; BiPAP, bilevel positive airway pressure; IPAP, inspiratory positive airway pressure; EPAP, expiratory positive airway pressure.

### Study quality evaluation

The risk of bias of the included studies was assessed using the Cochrane RoB 2.0 tool, and the results are summarized in Table 2. Overall, the methodological quality of the included trials was acceptable, with no study judged as having a high risk of bias. The randomization process was considered low risk in all studies because appropriate methods such as computer-generated randomization, block randomization, random number tables, centralized randomization, or sealed opaque envelopes were reported. All studies used open-label designs because blinding of respiratory support interventions was practically difficult. Therefore, the domain of deviations from intended interventions was judged as having some concerns in six studies (22–24, 26–28). However, four studies used intention-to-treat analyses or predefined objective escalation criteria, which likely reduced the impact of performance bias (25, 29–31). The risk of bias due to missing outcome data was low for all the included studies because follow-up was complete or nearly complete across studies, with balanced exclusions or no significant missing data. Outcome measurement was considered low risk in five studies because treatment failure and invasive MV were defined using prespecified objective criteria, including changes in respiratory rate, oxygen saturation, blood gas parameters, apnea episodes, or escalation of respiratory support (22, 23, 25, 29, 31). However, the other five studies (24, 26–28, 30) were judged as having some concerns in this domain because subjective clinical symptom scores or physician judgment contributed partly to treatment failure assessment. All studies were considered low risk for selective reporting because prespecified outcomes were adequately reported and several studies were prospectively registered. Overall, three studies (25, 29, 31) were judged as low risk of bias overall, whereas the remaining studies (22–24, 26–28, 30) were judged as having some concerns, mainly related to the unavoidable open-label design and partial reliance on subjective clinical assessments.

**Table 2:** Study quality evaluation via the RoB 2.0 tool.

Study	Randomization process	Deviations from intended interventions	Missing outcome data	Measurement of the outcome	Selection of the reported results	Overall
Milesi 2017	Low risk (Central randomization with block sizes of 2 and 4, stratified by center. Allocation concealed in opaque sealed envelopes)	Some concerns [Open-label design (no blinding of personnel or participants due to nature of interventions). However, failure criteria were prespecified and objective. Intention-to-treat analysis performed]	Low risk [Primary outcome reported for all 142 randomized patients (no missing data). Follow-up complete]	Low risk [Treatment failure defined by prespecified objective criteria (mWCAS, RR, EDIN score, apnea episodes). Outcomes assessed systematically]	Low risk [Primary outcome prespecified (NCT02457013). All prespecified outcomes reported. No evidence of selective reporting]	Some concerns
Ji 2017	Low risk (Random number table used with assignment; Adequate sequence generation)	Some concerns [Open-label design (no blinding of personnel or participants). However, objective outcomes (blood gas, intubation) less susceptible to bias. Intention-to-treat not explicitly stated but appears maintained]	Low risk (Complete follow-up reported for all 32 patients. No missing outcome data)	Low risk [Predefined criteria of outcomes; objective outcomes (blood gas, intubation) are low risk]	Low risk (All prespecified outcomes reported. No evidence of selective reporting)	Some concerns
Sarkar 2018	Low risk (Computer-generated block randomization schedule used on-site. Adequate sequence generation)	Some concerns [Open-label design (no blinding of personnel or participants)—stated as “open-label” pilot study. However, intention-to-treat analysis used with worst-case scenario for dropouts]	Low risk (Complete follow-up for all 31 randomized patients. Figure 1 CONSORT diagram shows no dropout after randomization)	Some concerns [Primary outcome (reduction of MV need) includes subjective components (COMFORT score, RDAI) that may be influenced by lack of blinding. Objective measures (SpO_2_, blood gases) are low risk]	Low risk (All prespecified outcomes reported at multiple time points. No evidence of selective reporting)	Some concerns
Vahlkvist 2020	Low risk (Randomized trial with computer-generated allocation)	Some concerns [Open-label design (no blinding of personnel or participants). However, intention-to-treat principles likely followed. Protocol amendment approved for third season]	Low risk (Dropouts reported and relatively balanced. Analysis uses appropriate methods, with random intercept model)	Some concerns [Primary outcomes (pCO_2_, RR) are objective—low risk. mWCAS and pain scores (NIPS/FLACC) are subjective and may be influenced by lack of blinding]	Low risk (All prespecified outcomes reported. Trial registered prospectively. No evidence of selective reporting)	Some concerns
Li 2020	Low risk (Random number table used for group assignment. Adequate sequence generation)	Some concerns (Open-label design, no blinding of personnel or participants)	Low risk (Complete follow-up reported for all patients. No missing outcome data)	Some concerns [Subjective outcomes (CSS score, cough disappearance, rales disappearance) may be influenced by lack of blinding. Objective outcomes (respiratory rate, TeSO_2_, PaO_2_) are low risk])	Low risk (All prespecified outcomes reported. No evidence of selective reporting)	Some concerns
Cesar 2020	Low risk (Opaque envelope method with 1:1 allocation. Nursing staff not involved in study performed randomization)	Low risk [Open-label design (unavoidable due to nature of interventions). No crossover between groups—patients who failed were escalated to BiPAP or intubation, not switched to alternative study treatment. Intention-to-treat analysis preserved]	Low risk (Complete follow-up for all 63 patients. Primary and secondary outcomes reported for all enrolled patients)	Low risk [Primary outcome (need for BiPAP or intubation) is objective and clinically meaningful—less susceptible to bias than composite scores. Escalation decisions made by care team based on clinical deterioration]	Low risk (All prespecified outcomes reported. No evidence of selective reporting)	Low risk
Zhang 2020	Low risk (Random number table used for group assignment)	Some concerns (Open-label design; no mention of intention-to-treat analysis)	Low risk (Complete data reported for 100 patients)	Some concerns (subjective clinician judgment based on symptom scores)	Low risk (All prespecified outcomes reported)	Some concerns
Qi 2021	Low risk (Random number table used for group assignment)	Low risk [Open-label design (unavoidable). Intention-to-treat appears maintained]	Low risk (Complete data reported for all 84 patients. No dropouts mentioned)	Some concerns [Treatment failure is based on subjective criteria (wheezing, retractions, mental status, TeSO_2_). However, TeSO_2_ > 95% is an objective threshold, and failure led to explicit treatment change]	Low risk (All prespecified outcomes reported. No evidence of selective reporting)	Some concerns
Borgi 2021	Low risk (Block randomization with blocks of four; randomization lists prepared by epidemiology unit; allocation based on identification number in block)	Low risk [Open-label design (unavoidable). Per-protocol analysis used]	Low risk [13/268 (4.9%) excluded after randomization for predefined exclusion criteria (pertussis, recurrent wheezing, congenital heart defect). Exclusions were not related to treatment outcome. Complete data for 255 analyzed patients]	Low risk [Primary outcome (treatment success = no need for care escalation) is clearly defined with objective criteria (FiO_2_ > 60% to maintain SpO_2_ ≤ 90% OR increasing WOB). Escalation decisions based on objective thresholds]	Low risk [All prespecified outcomes reported. Trial registered at ClinicalTrials.gov (NCT04650230). No evidence of selective reporting]	Low risk
Maya 2024	Low risk [Web-based computer-generated unstratified block randomization (block sizes 4,6,8); sequentially numbered opaque sealed envelopes; person not involved in study performed allocation]	Low risk [Open-label (unblinded intervention)—unavoidable. However, data analyst and statistician blinded until final analysis. Intention-to-treat analysis used. No protocol violations. Crossover allowed but none occurred]	Low risk [118 randomized, 118 analyzed (no missing outcome data). 10 declined consent before randomization—not a post-randomization issue]	Low risk [Primary outcome (treatment failure) uses objective criteria: 1-point mWCAS increase (validated score), RR rise >10 bpm, and escalation of support. Escalation decisions made by treating team but based on objective thresholds]	Low risk [All prespecified outcomes reported. Trial registered prospectively (CTRI/2019/07/020402). No evidence of selective reporting]	Low risk

RoB 2.0, Revised Cochrane risk-of-bias tool for randomized trials; RR, respiratory rate; EDIN, Échelle Douleur Inconfort Nouveau-Né scale; mWCAS, modified Wood's Clinical Asthma Score; SpO_2_, peripheral oxygen saturation; RDAI, Respiratory Distress Assessment Instrument; NIPS, Neonatal Infant Pain Scale; FLACC, Face, Legs, Activity, Cry, Consolability scale; CSS, clinical symptom score; TeSO_2_, transcutaneous oxygen saturation; PaO_2_, partial pressure of arterial oxygen; BiPAP, bilevel positive airway pressure; WOB, work of breathing; FiO_2_, fraction of inspired oxygen; PICU, pediatric intensive care unit; CTRI, Clinical Trials Registry of India.

### Primary outcome: HFNC vs. CPAP on treatment failure

**Figure 2:**
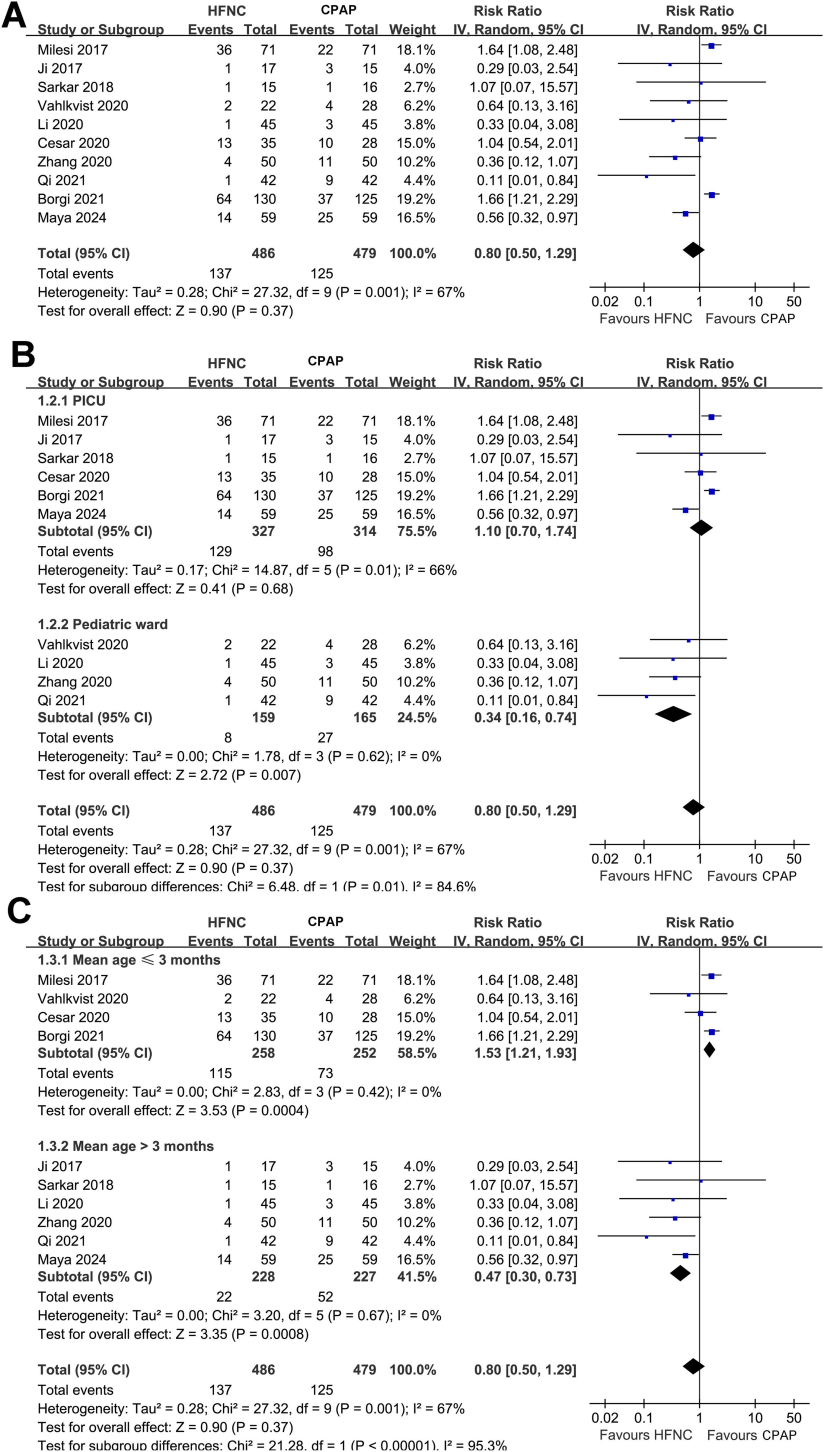
Forest plots for the meta-analysis comparing the rate of treatment failure in children with bronchiolitis receiving HFNC and CPAP. **(A)** overall meta-analysis; **(B)** subgroup analysis according to the clinical settings; and **(C)** subgroup analysis according to the mean ages of the children.

The pooled results of 10 RCTs (22–31) showed that overall, the rate of treatment failure was not significantly different between children with bronchiolitis receiving HFNC or CPAP (RR: 0.80, 95% CI: 0.50–1.29, *p* = 0.37; Figure 2A), with moderate heterogeneity (Cochrane Q test *p* = 0.001, *I*^2^ = 67%). Sensitivity analyses, performed by sequentially excluding individual studies, yielded consistent results (RR range: 0.60–0.90; all *p* > 0.05). An additional sensitivity analysis restricted to the five studies judged to have a low risk of bias in the outcome measurement domain (22, 23, 25, 29, 31) also yielded consistent findings (RR: 1.09, 95% CI: 0.67–1.78, *p* = 0.71; *I*^2^ = 73%).

**Figure 3:**
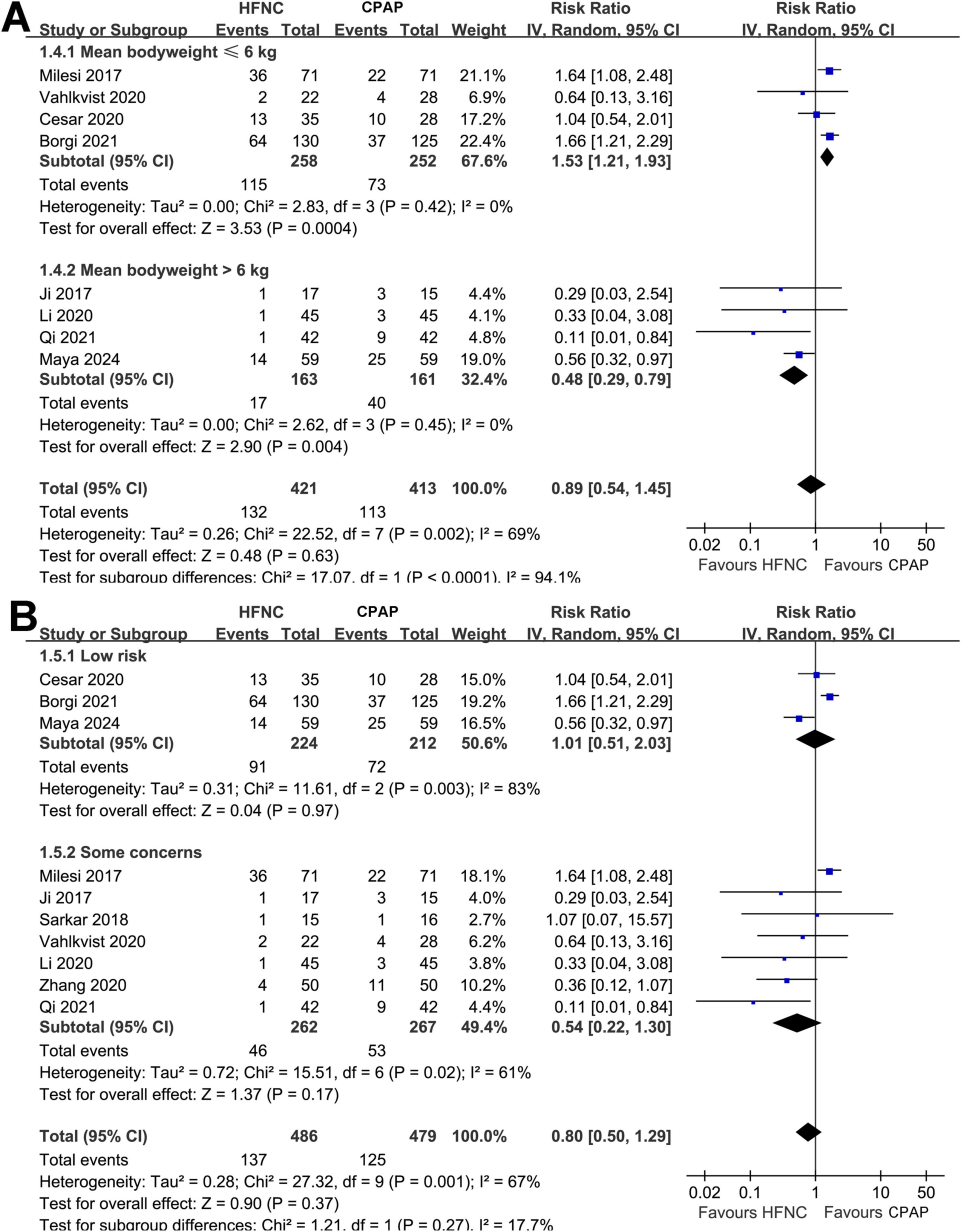
Forest plots for the subgroup analyses comparing the rate of treatment failure in children with bronchiolitis receiving HFNC and CPAP. **(A)** subgroup analysis according to the mean bodyweight of the children; and **(B)** subgroup analysis according to the study quality of the included RCTs.

Further subgroup analysis showed the rate of treatment failure was not significantly different between children with bronchiolitis receiving HFNC or CPAP in PICU, while HFNC was associated with a reduced risk of treatment failure in children with bronchiolitis treated in pediatric ward (RR: 1.10 vs. 0.34, *p* for subgroup difference = 0.01; Figure 2B). In addition, compared to CPAP, HFNC was associated with a reduced risk of treatment failure in children with mean ages > 3 months, but not in children with mean ages ≤3months (RR: 0.47 vs. 1.53, *p* for subgroup difference <0.001; Figure 2C). Similarly, HFNC was associated with a reduced risk of treatment failure in children with mean body weight >6 kg, but not in children with mean body weight ≤6 kg (RR: 0.48 vs. 1.53, *p* for subgroup difference < 0.001; Figure 3A). No significant subgroup difference was observed between studies with low risk of bias according to RoB 2.0 and those with some concerns (RR: 1.01 vs. 0.54, *p* for subgroup difference = 0.27; Figure 3B).

Univariate meta-regression analyses were performed to explore potential sources of heterogeneity for the primary outcome of treatment failure (Table 3). Mean age of the children (coefficient = −0.13, 95% CI: −0.23 to −0.03, *p* = 0.02) and mean body weight (coefficient = −0.40, 95% CI: −0.65 to −0.16, *p* = 0.01) were both significantly associated with the effect estimates, explaining a substantial proportion of between-study variance (adjusted *R*^2^ = 100.0% and 85.1%, respectively). Specifically, studies including older or heavier children tended to show a lower RR of treatment failure with HFNC compared with CPAP. In contrast, sample size and proportion of boys were not significantly associated with the effect estimates (both *p* > 0.05). These findings suggest that age and body weight may partly contribute to the observed heterogeneity among studies.

**Table 3:** Results of univariate meta-regression analysis.

Variables	RR comparing the risk of treatment failure of children with bronchiolitis treated with HFNC vs. CPAP			
	Coefficient	95% CI	*p* values	Adjusted R^2^
Sample size	0.0052	−0.0007 to 0.0112	0.09	45.1%
Mean age (months)	−0.13	−0.23 to −0.03	0.02	100.0%
Boy (%)	0.012	−0.121 to 0.144	0.84	0%
Mean body weight (kg)	−0.40	−0.65 to −0.16	0.01	85.1%

RR, risk ratio; HFNC, high-flow nasal cannula; CPAP, continuous positive airway pressure; CI, confidence interval; *R*^2^, coefficient of determination.

### Secondary outcome: HFNC vs. CPAP on invasive MV and AEs

**Figure 4:**
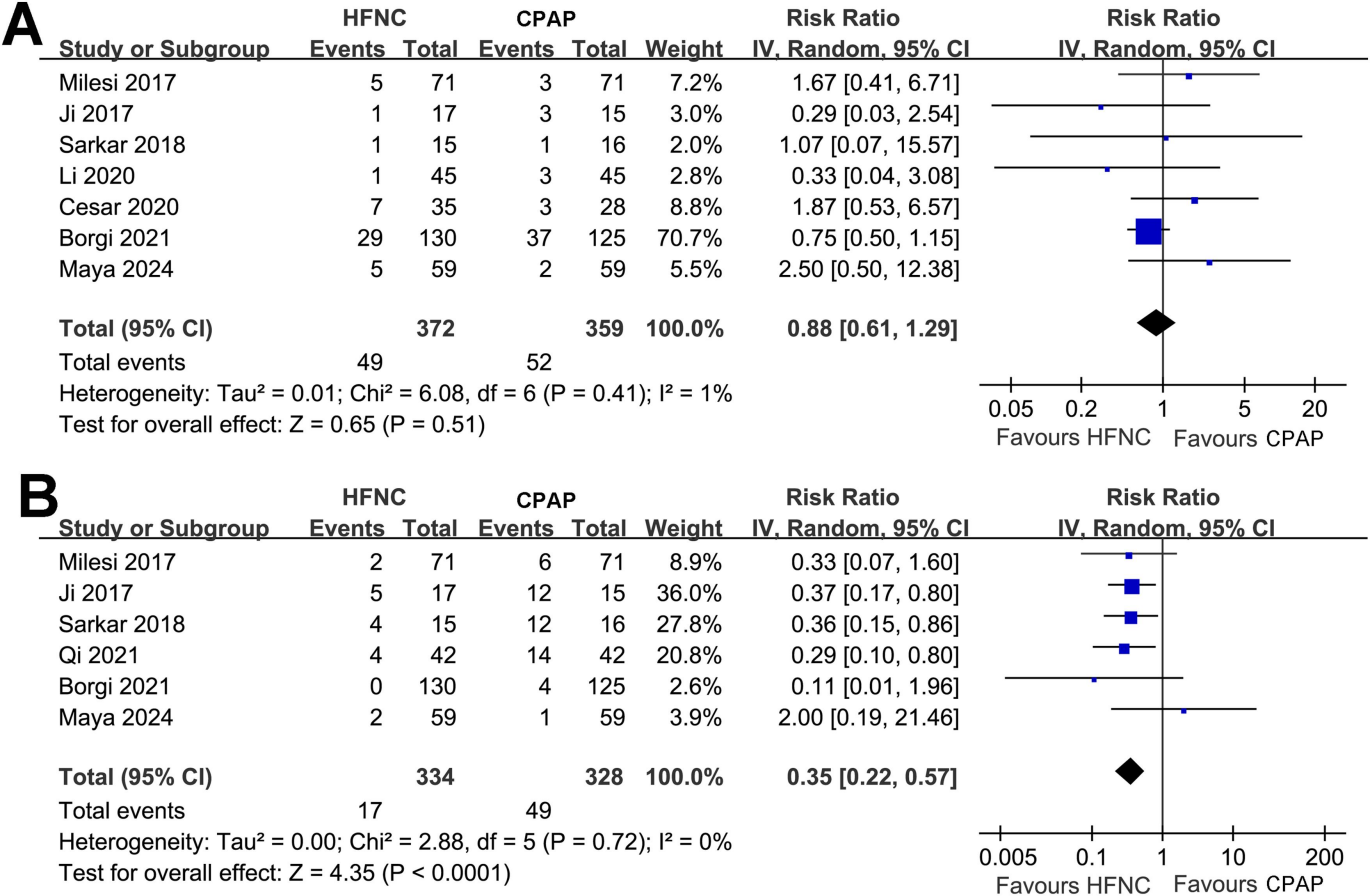
Forest plots for the meta-analysis comparing the rate of invasive MV and the incidence of overall AEs in children with bronchiolitis receiving HFNC and CPAP. **(A)** meta-analysis for the rate of invasive MV; and **(B)** meta-analysis for the incidence of overall AEs.

The pooled results of seven (22–26, 29, 31) RCTs did not suggest a significantly different rate of invasive MV in children with bronchiolitis treated with HFNC and CPAP (RR: 0.88, 95% CI: 0.61–1.29, *p* = 0.51; Figure 4A), with mild heterogeneity (Cochrane Q test *p* = 0.41, *I*^2^ = 1%). Sensitivity analyses, performed by sequentially excluding individual studies, yielded consistent results (RR range: 0.81–1.29; all *p* > 0.05). The incidence of AEs was reported in six (22–24, 29–31) studies. Overall, AEs were generally uncommon in both groups, although HFNC tended to be associated with a lower incidence of device-related complications compared with CPAP (RR: 0.35, 95% CI: 0.22–0.57, *p* < 0.001; Figure 4B) with no significant heterogeneity (Cochrane Q test *p* = 0.72, *I*^2^ = 0%). Results of sensitivity analysis by excluding one study at a time showed consistent results (RR: 0.33–0.38, all *p* < 0.05). The reported AEs mainly included nasal trauma or skin injury, feeding difficulty requiring nasogastric tube placement, nasal septum injury, hypercapnia, air leak, and nosocomial infection. Most AEs were mild and related to the nasal interface used for respiratory support.

### Publication bias

**Figure 5:**
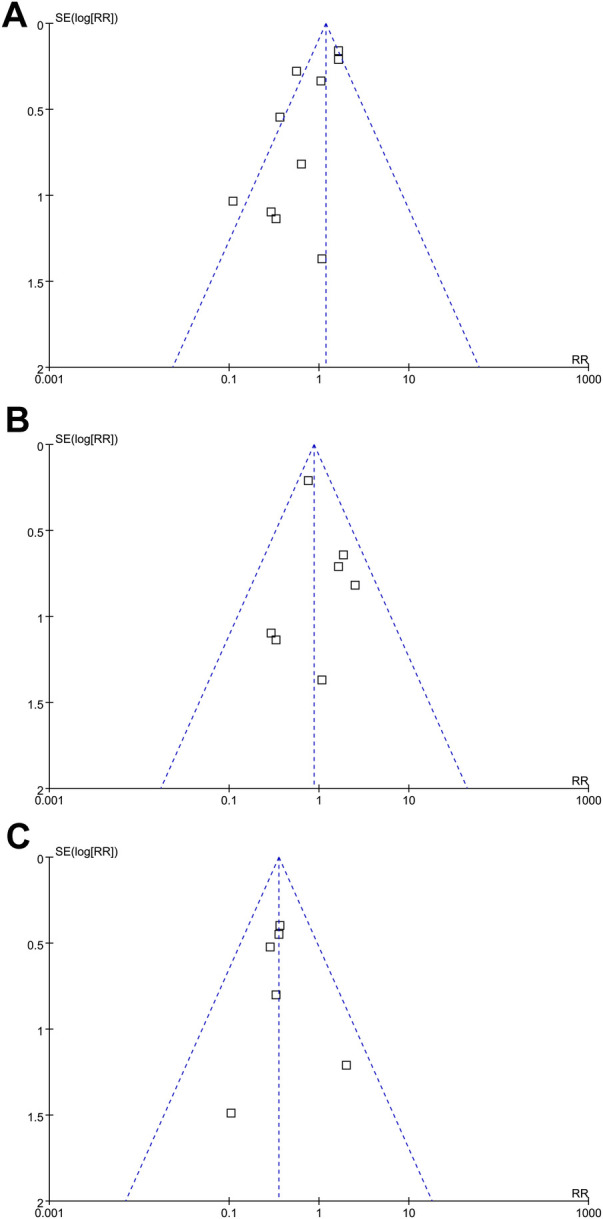
Funnel plots evaluating the publication bias underlying the meta-analyses. **(A)** funnel plots for the meta-analysis of treatment failure; **(B)** funnel plots for the meta-analysis of invasive MV; and **(C)** funnel plots for the meta-analysis of overall AEs.

Funnel plots assessing publication bias for the meta-analyses comparing HFNC and CPAP on treatment failure, invasive MV, and AEs are shown in Figures 5A–C. Visual inspection suggested an approximately symmetrical distribution of the included studies, indicating a low likelihood of publication bias. This was further supported by Egger's regression tests, which did not detect significant asymmetry (*p* = 0.18, 0.61, and 0.52, respectively). However, these findings should be interpreted with caution, as the number of included studies was relatively small (k = 10, 7, and 6, respectively), which may limit the statistical power to detect publication bias.

### Certainty of evidence

The certainty of evidence for the primary outcome was assessed using the GRADE approach (Table 4). The certainty of evidence for treatment failure was rated as moderate according to the GRADE approach, mainly due to imprecision because the pooled estimate was close to null and the 95% CI crossed no effect. In contrast, the certainty of evidence for invasive MV and overall AEs was rated as low because of imprecision and the limited number of included studies. Risk of bias, inconsistency, and indirectness were generally not considered serious across outcomes, as the included RCTs directly addressed the target population and interventions, and the effect estimates were generally consistent among studies. Publication bias was not evident based on funnel plot assessment, although the relatively small number of studies warrants cautious interpretation of the findings.

**Table 4:** Summary of findings and certainty of evidence (GRADE).

Outcome	No. of participants (studies)	Study design	Risk of bias	Inconsistency	Indirectness	Imprecision	Publication bias	Relative effect:	Certainty of evidence (GRADE)	Comments
Treatment failure	965 (10 RCTs)	RCTs	Not serious—Although all trials were open-label, this was unavoidable in such studies. In addition, no studies were judged as “high risk” in RoB 2.0	Not serious—Effect estimates were generally consistent across studies, with moderate heterogeneity (*I*^2^ = 67%), which was explained in subgroup and meta-regression analyses	Not serious—Included studies directly addressed the target population (children with bronchiolitis), intervention (HFNC), comparator (CPAP), and outcome (treatment failure)	Serious—The pooled estimate was close to null and the 95% CI crossed no effect (RR 0.80, 0.50–1.29), indicating uncertainty in the magnitude and direction of effect	Not serious—Funnel plot appeared approximately symmetrical, and no substantial publication bias was suggested	RR (95% CI): 0.80 (0.50–1.29)	⊕⊕⊕◯ Moderate	HFNC appears to confer similar rate of treatment failure compared to CPAP; however, the effect estimate is imprecise and further high-quality trials may influence the results
Rate of MV	731 (7 RCTs)	RCTs	Not serious—Although all trials were open-label, this was unavoidable in such studies. In addition, no studies were judged as “high risk” in RoB 2.0	Not serious–Effect estimates were generally consistent across studies, with mild heterogeneity (*I*^2^ = 1%).	Not serious–Included studies directly addressed the target population (children with bronchiolitis), intervention (HFNC), comparator (CPAP), and outcome (MV rate)	Serious—The pooled estimate was close to null and the 95% CI crossed no effect (RR 0.88, 0.61–1.29). Also only 7 studies were available, indicating uncertainty in the magnitude and direction of effect	Serious—Funnel plot appeared approximately symmetrical. However, the results should be interpreted cautiously in view of the limited studies included	RR (95% CI): 0.88 (0.61–1.29)	⊕⊕◯◯ Low	The rate of MV might be similar between those treated with HFNC and CPAP; however, the effect estimate is imprecise and further high-quality trials may influence the results
Overall AEs	662 (6 RCTs)	RCTs	Not serious—Although all trials were open-label, this was unavoidable in such studies. In addition, no studies were judged as “high risk” in RoB 2.0	Not serious—Effect estimates were generally consistent across studies, with mild heterogeneity (*I*^2^ = 0%).	Not serious—Included studies directly addressed the target population (children with bronchiolitis), intervention (HFNC), comparator (CPAP), and outcome (AEs)	Serious—Although the pooled CI excludes no effect, only 6 studies were available, indicating uncertainty in the magnitude and direction of effect	Serious—Funnel plot appeared approximately symmetrical. However, the results should be interpreted cautiously in view of the limited studies included	RR (95% CI): 0.35 (0.22–0.57)	⊕⊕◯◯ Low	CPAP might confer higher risk of overall AEs compared to HFNC; however, only limited studies were included and the results should be interpreted with caution

GRADE, Grading of Recommendations Assessment, Development and Evaluation; RCTs, randomized controlled trials; RoB 2.0, Revised Cochrane risk-of-bias tool for randomized trials; HFNC, high-flow nasal cannula; CPAP, continuous positive airway pressure; MV, mechanical ventilation; AEs, adverse events; RR, risk ratio; CI, confidence interval.

Specific reasons for each GRADE domain, including:

Risk of bias: Downgraded if a significant proportion of studies had unclear or high risk of bias in key domains (e.g., random sequence generation, allocation concealment, or selective reporting).

Inconsistency: Downgraded if substantial heterogeneity was observed (*I*^2^ > 75%) and could not be explained by subgroup analyses or meta-regression.

Indirectness: Evaluated but not downgraded, as all included studies directly assessed the population and outcomes of interest.

Imprecision: Downgraded if confidence intervals were wide, or if the overall sample size was small.

Publication bias: Assessed using funnel plots and Egger's test; downgraded if significant asymmetry suggested potential bias.

## Discussion

In this systematic review and meta-analysis of 10 RCTs involving 965 children with bronchiolitis, we found that HFNC and CPAP provided overall comparable efficacy regarding treatment failure and invasive MV, while HFNC was associated with fewer AEs. However, subgroup and meta-regression analyses suggested that the relative efficacy of HFNC may vary according to patient characteristics and clinical settings. Specifically, HFNC appeared to be associated with a lower risk of treatment failure in studies including older and heavier children and in pediatric ward settings, whereas CPAP appeared to be more favorable in younger and lighter infants treated in PICUs. Therefore, although HFNC and CPAP appear to provide comparable overall efficacy, our subgroup and meta-regression analyses suggest that their relative effectiveness may vary according to patient characteristics, including age, body weight, and clinical setting, which may partially reflect differences in disease severity. However, because disease severity was not directly analyzed and these findings were derived from study-level data, they should be considered exploratory and hypothesis-generating. Individualized respiratory support strategies based on patient characteristics may therefore help optimize clinical outcomes.

The comparable efficacy of HFNC and CPAP observed in the overall analysis is biologically plausible because both modalities improve oxygenation and reduce respiratory effort through the provision of positive airway pressure and improved gas exchange. HFNC delivers heated humidified gas at high flow rates, which can wash out nasopharyngeal dead space, reduce inspiratory resistance, improve mucociliary function, and generate a certain degree of PEEP (32, 33). These physiological effects may alleviate airway obstruction and decrease work of breathing in bronchiolitis (8). CPAP, in contrast, provides more stable and continuous airway distending pressure throughout the respiratory cycle, thereby improving functional residual capacity, preventing airway collapse, and reducing inspiratory effort more effectively in severe respiratory distress (12, 34). Accordingly, CPAP may theoretically provide greater respiratory support in infants with more severe bronchiolitis, whereas HFNC may offer adequate support in milder disease while improving comfort and tolerance (35, 36). This interpretation is also supported by the significantly lower incidence of AEs with HFNC in our analysis. Most AEs associated with CPAP were device-related complications, including nasal trauma, septal injury, and skin lesions, which are likely related to the tighter-fitting interfaces and higher pressure delivery required for CPAP therapy (37).

The subgroup and meta-regression findings regarding age and body weight are clinically interesting and may reflect developmental differences in respiratory physiology and disease severity among infants with bronchiolitis. Younger and lighter infants generally have narrower airways, lower functional residual capacity, reduced respiratory reserve, and higher susceptibility to airway collapse and severe respiratory distress (38). In these children, the more stable and continuous airway pressure generated by CPAP may provide greater physiological benefit than HFNC (39). In contrast, older and heavier infants may tolerate bronchiolitis better because of relatively larger airway caliber, improved respiratory muscle strength, and more mature pulmonary mechanics, thereby allowing HFNC to provide sufficient respiratory support through dead-space washout and moderate airway pressure generation (38, 40). In addition, older infants may better tolerate the HFNC interface, remain less agitated during therapy, and require fewer interruptions related to discomfort, which could contribute to more effective and sustained respiratory support in routine clinical practice. Conversely, the tighter-fitting CPAP interface may offer less incremental physiological benefit once adequate airway support can be achieved with HFNC, while potentially being associated with greater discomfort and interface-related complications. The consistency between subgroup analyses and meta-regression findings further strengthens the potential relevance of these factors as effect modifiers. Importantly, these findings are also broadly consistent with previous studies evaluating predictors of HFNC success in bronchiolitis, which have suggested that younger age, lower body weight, severe respiratory distress, hypercapnia, and PICU admission are associated with higher rates of HFNC failure and escalation of respiratory support (41–44). Similarly, the apparent advantage of HFNC in pediatric ward settings may reflect differences in disease severity rather than the respiratory support modality itself, as children managed outside the PICU generally have less severe illness and may derive sufficient benefit from the simpler, better-tolerated HFNC system. However, these subgroup findings were derived from study-level analyses and should be considered exploratory rather than causal, as residual confounding and ecological bias cannot be excluded. Future individual participant data meta-analyses and adequately powered randomized trials are warranted to determine whether age, body weight, and clinical setting truly modify the comparative effectiveness of HFNC and CPAP.

The observed differences according to clinical setting may also reflect differences in disease severity and patient selection. Children managed in PICUs are generally more severely ill, with greater respiratory distress, hypoxemia, or risk of respiratory failure compared with those treated in pediatric wards. In this context, the more stable positive airway pressure generated by CPAP may be advantageous in preventing worsening respiratory failure and treatment escalation (45). Conversely, children treated in pediatric wards may represent a less severe population in whom HFNC provides sufficient respiratory support while offering practical advantages such as easier application, improved comfort, reduced nursing burden, and fewer interface-related complications (46). In addition, pediatric ward settings may favor HFNC because of its relative simplicity and lower monitoring requirements compared with CPAP (47). However, these subgroup findings should be interpreted cautiously because they were based on study-level rather than individual patient-level analyses, and the subgroup categories partly overlap with differences in age and body weight among studies.

Compared with previous meta-analyses (13–16), the present study has several important strengths. First, this is one of the most up-to-date meta-analyses on this topic and included recently published RCTs that were not incorporated in earlier reviews. Second, unlike some previous meta-analyses that included retrospective studies, the current study included RCTs only, thereby reducing the potential influence of selection bias and confounding. Third, this study included Chinese-language RCTs identified through Wanfang and CNKI, which expanded the evidence base and may reduce language-related publication bias. Fourth, we comprehensively evaluated multiple clinically relevant outcomes, including treatment failure, invasive MV, and AEs. Fifth, we performed subgroup, sensitivity, and meta-regression analyses to explore potential sources of heterogeneity and identify possible effect modifiers. Finally, the certainty of evidence was evaluated using the GRADE approach, which improves the interpretability and clinical relevance of the findings.

Several limitations of this meta-analysis should also be acknowledged. First, although all currently available RCTs were included, the overall sample size remained relatively modest for a clinically heterogeneous condition such as bronchiolitis. In addition, heterogeneity in the definition of treatment failure represents an important limitation. Although treatment failure generally reflected worsening respiratory distress or escalation of respiratory support, the specific criteria varied across studies and included combinations of clinical symptoms, oxygenation parameters, blood gas abnormalities, physician judgment, or need for transfer to intensive care. Together with differences in clinical settings, patient characteristics, and respiratory support protocols, this variability may have contributed to between-study heterogeneity and reduced the ability to detect true differences between HFNC and CPAP. Nevertheless, an additional sensitivity analysis restricted to studies judged to have a low risk of bias in the outcome measurement domain yielded results consistent with the primary analysis, supporting the robustness of our findings. Second, all analyses were based on study-level data rather than individual participant data (IPD), which limited our ability to adjust for important patient-level confounders such as baseline respiratory severity, comorbidities, hypercapnia, prematurity, or RSV status. In addition, because treatment outcomes were not reported separately for RSV-positive and non-RSV bronchiolitis in the included trials, we were unable to evaluate whether the comparative effectiveness of HFNC and CPAP differed according to viral etiology. Third, considerable variability existed regarding HFNC and CPAP protocols, including the clinical criteria used to initiate non-invasive respiratory support, flow rates, pressure settings, interfaces, FiO_2_ titration, escalation criteria, and weaning strategies. In addition, pre-randomization supportive and pharmacological management was not consistently reported or standardized, precluding assessment of its potential influence on treatment outcomes. Fourth, although all included studies were randomized, most used open-label designs because blinding was practically difficult for respiratory support interventions. Fifth, mortality data were extremely limited because only one included study (29) reported all-cause mortality, thereby preventing meaningful pooled analysis for this outcome. Sixth, data regarding long-term clinical outcomes, healthcare costs, patient comfort, feeding tolerance, and neurodevelopmental consequences were unavailable. Finally, the number of studies included in some subgroup analyses remained limited, and the subgroup findings should therefore be considered exploratory and hypothesis-generating rather than definitive.

From a clinical perspective, the present findings suggest that both HFNC and CPAP are reasonable noninvasive respiratory support options for children with bronchiolitis, and the choice between these modalities should be individualized according to the patient's clinical condition, treatment tolerance, local expertise, and resource availability. Our subgroup and meta-regression analyses further suggest that the comparative effectiveness of HFNC and CPAP may vary according to patient age, body weight, and clinical setting, which may partially reflect differences in disease severity. Accordingly, HFNC may be considered an appropriate initial respiratory support strategy for selected older or heavier children managed in pediatric ward settings because of its favorable tolerability and lower incidence of adverse events, whereas CPAP may remain a reasonable option for younger infants or those with more severe respiratory distress requiring PICU management. However, because these observations were derived from study-level analyses, no validated age or body weight thresholds can currently be recommended, and these findings should be regarded as exploratory and hypothesis-generating rather than definitive.

Nevertheless, the overall conclusions should be interpreted cautiously in light of the moderate-certainty evidence for treatment failure and the low-certainty evidence for invasive mechanical ventilation and adverse events, as assessed using the GRADE approach. Therefore, the available evidence remains insufficient to recommend one modality as universally superior. Future large-scale multicenter RCTs with standardized definitions of treatment failure and harmonized respiratory support protocols are needed to clarify the optimal selection of HFNC and CPAP in different clinical scenarios. Individual participant data meta-analyses will also be important to determine whether patient characteristics, including age, body weight, disease severity, and RSV status, truly modify the comparative effectiveness of these respiratory support strategies. In addition, future studies should evaluate long-term clinical outcomes, cost-effectiveness, patient comfort, feeding outcomes, and healthcare resource utilization associated with these interventions.

## Conclusions

In conclusion, the present meta-analysis suggests that HFNC and CPAP provide overall comparable efficacy for noninvasive respiratory support in children with bronchiolitis, while HFNC may reduce device-related adverse events. Subgroup and meta-regression analyses further suggest that the comparative effectiveness of HFNC and CPAP may vary according to patient age, body weight, and clinical setting, with relatively more favorable outcomes observed in older and heavier children and in pediatric ward settings. However, these subgroup findings should be considered exploratory and hypothesis-generating. Further high-quality multicenter RCTs and individual participant data meta-analyses are warranted to confirm these findings and help refine individualized respiratory support strategies for bronchiolitis.

## Data Availability

The original contributions presented in the study are included in the article/Supplementary Material, further inquiries can be directed to the corresponding author/s.
