# Supplementary material for: High-flow nasal cannula versus continuous positive airway pressure for noninvasive respiratory support in children with bronchiolitis: a meta-analysis of randomized controlled trials
Source: Front Pediatr. 2026 Aug 5;14:1892131. doi: 10.3389/fped.2026.1892131 (PMC13486297; doi:10.3389/fped.2026.1892131)
Supplement: Supplementary file 1 [file Supplementaryfile1.docx]

**Detailed search strategy for each database**

**PubMed**

("Bronchiolitis"[Mesh] OR bronchiolitis[Title/Abstract] OR "viral bronchiolitis"[Title/Abstract] OR RSV[Title/Abstract] OR "Respiratory Syncytial Viruses"[Mesh] OR "respiratory syncytial virus"[Title/Abstract]) AND ("High-Flow Nasal Cannula"[Title/Abstract] OR "high-flow nasal cannula"[Title/Abstract] OR "high flow nasal cannula"[Title/Abstract] OR HFNC[Title/Abstract] OR "high-flow oxygen"[Title/Abstract] OR HHHFNC[Title/Abstract] OR HHHF[Title/Abstract]) AND ("Continuous Positive Airway Pressure"[Mesh] OR CPAP[Title/Abstract] OR "nasal CPAP"[Title/Abstract] OR "bubble CPAP"[Title/Abstract] OR NIV[Title/Abstract] OR NIPPV[Title/Abstract] OR "noninvasive ventilation"[Title/Abstract] OR "non-invasive ventilation"[Title/Abstract] OR "noninvasive positive pressure ventilation"[Title/Abstract]) AND (randomized[Title/Abstract] OR randomised[Title/Abstract] OR randomly[Title/Abstract] OR trial[Title/Abstract] OR RCT[Title/Abstract] OR control[Title/Abstract] OR controlled[Title/Abstract] OR allocated[Title/Abstract] OR assigned[Title/Abstract] OR "Randomized Controlled Trial"[Publication Type])

**Cochrane Library**

**Cochrane Library**

(MeSH descriptor: [Bronchiolitis] explode all trees OR bronchiolitis:ti,ab,kw OR "viral bronchiolitis":ti,ab,kw OR RSV:ti,ab,kw OR "respiratory syncytial virus":ti,ab,kw) AND ("high-flow nasal cannula":ti,ab,kw OR "high flow nasal cannula":ti,ab,kw OR HFNC:ti,ab,kw OR "high-flow oxygen":ti,ab,kw OR HHHFNC:ti,ab,kw OR HHHF:ti,ab,kw) AND (MeSH descriptor: [Continuous Positive Airway Pressure] explode all trees OR CPAP:ti,ab,kw OR "nasal CPAP":ti,ab,kw OR "bubble CPAP":ti,ab,kw OR NIV:ti,ab,kw OR NIPPV:ti,ab,kw OR "noninvasive ventilation":ti,ab,kw OR "non-invasive ventilation":ti,ab,kw OR "noninvasive positive pressure ventilation":ti,ab,kw) AND (randomized:ti,ab,kw OR randomised:ti,ab,kw OR randomly:ti,ab,kw OR trial:ti,ab,kw OR RCT:ti,ab,kw OR control:ti,ab,kw OR controlled:ti,ab,kw OR allocated:ti,ab,kw OR assigned:ti,ab,kw)

**Embase**

('bronchiolitis'/exp OR bronchiolitis:ti,ab OR 'viral bronchiolitis':ti,ab OR RSV:ti,ab OR 'respiratory syncytial virus'/exp OR 'respiratory syncytial virus':ti,ab) AND ('high flow nasal cannula'/exp OR 'high-flow nasal cannula':ti,ab OR 'high flow nasal cannula':ti,ab OR HFNC:ti,ab OR 'high-flow oxygen':ti,ab OR HHHFNC:ti,ab OR HHHF:ti,ab) AND ('continuous positive airway pressure'/exp OR CPAP:ti,ab OR 'nasal CPAP':ti,ab OR 'bubble CPAP':ti,ab OR NIV:ti,ab OR NIPPV:ti,ab OR 'noninvasive ventilation':ti,ab OR 'non-invasive ventilation':ti,ab OR 'noninvasive positive pressure ventilation':ti,ab) AND (randomized:ti,ab OR randomised:ti,ab OR randomly:ti,ab OR trial:ti,ab OR RCT:ti,ab OR control:ti,ab OR controlled:ti,ab OR allocated:ti,ab OR assigned:ti,ab OR 'randomized controlled trial'/exp)

**Web of Science**

TS=(bronchiolitis OR "viral bronchiolitis" OR RSV OR "respiratory syncytial virus")

AND TS=("high-flow nasal cannula" OR "high flow nasal cannula" OR HFNC OR "high-flow oxygen" OR HHHFNC OR HHHF) AND TS=(CPAP OR "nasal CPAP" OR "bubble CPAP" OR NIV OR NIPPV OR "noninvasive ventilation" OR "non-invasive ventilation" OR "noninvasive positive pressure ventilation") AND TS=(randomized OR randomised OR randomly OR trial OR RCT OR control OR controlled OR allocated OR assigned)

**Wanfang**

主题 = ((“毛细支气管炎”OR “病毒性毛细支气管炎”OR RSVOR “呼吸道合胞病毒”) AND (“高流量鼻导管”OR “经鼻高流量”OR “加温湿化高流量”OR HFNC OR HHHFNC OR HHHF) AND (CPAP OR “持续气道正压通气”OR “经鼻持续气道正压通气”OR “气泡CPAP”OR NIVOR NIPPVOR “无创通气”OR “无创正压通气”) AND (“随机”OR “随机对照”OR “对照”OR “试验”))

**CNKI**

SU = ((“毛细支气管炎”OR “病毒性毛细支气管炎”OR RSV OR “呼吸道合胞病毒”) AND (“高流量鼻导管”OR “经鼻高流量”OR “加温湿化高流量”OR HFNC OR HHHFNC OR HHHF) AND (CPAP OR “持续气道正压通气”OR “经鼻持续气道正压通气”OR “气泡CPAP”OR NIV OR NIPPV OR “无创通气”OR “无创正压通气”) AND (“随机”OR “随机对照”OR “对照”OR “试验”))
